# Emerging Risk Factors and Prevention of Perioperative Pulmonary Complications

**DOI:** 10.1155/2014/546758

**Published:** 2014-01-21

**Authors:** Priyanka Bhateja, Roop Kaw

**Affiliations:** ^1^Department of Hospital Medicine (Medicine Institute), Cleveland Clinic Foundation, Cleveland, OH 44195, USA; ^2^Department of Hospital Medicine (Medicine Institute) and Outcomes Research (Anesthesiology Institute), Cleveland Clinic Lerner College of Medicine, Desk A13, 9500 Euclid Avenue, Cleveland, OH 44195, USA

## Abstract

Modern surgery is faced with the emergence of newer “risk factors” and the challenges associated with identifying and managing these risks in the perioperative period. Obstructive sleep apnea and obesity hypoventilation syndrome pose unique challenges in the perioperative setting. Recent studies have identified some of the specific risks arising from caring for such patients in the surgical setting. While all possible postoperative complications are not yet fully established or understood, the prevention and management of these complications pose even greater challenges. Pulmonary hypertension with its changing epidemiology and novel management strategies is another new disease for the surgeon and the anesthesiologist in the noncardiac surgical setting. Traditionally most such patients were not considered surgical candidates for any required elective surgery. Our review discusses these disease entities which are often undiagnosed before elective noncardiac surgery.

## 1. Introduction

Medical literature abounds with wide recognition and awareness of cardiac risk factors and their effective management in modifying perioperative morbidity and mortality. Although large cohort studies have shown similar rates of postoperative pulmonary and cardiac complications in patients undergoing noncardiac surgery (NCS), there are no well-known risk indices that can help predict and manage postoperative pulmonary complications from underlying pulmonary disease [1–3]. Guidelines from the American College of Physicians guide list American society of anesthesia (ASA) class 2 or higher, chronic obstructive pulmonary disease, impaired functional class, and congestive heart failure as patient-related risk factors for postoperative pulmonary complications [4, 5]. Obstructive sleep apnea (OSA), obesity hypoventilation syndrome (OHS), and pulmonary hypertension (PH) are gaining increasing recognition as pulmonary risk factors for patients undergoing noncardiac surgery (NCS). This paper presents an overview of recognition and management of these important emerging risk factors in the perioperative period.

## 2. Obstructive Sleep Apnea

### 2.1. Scope of the Problem in Perioperative Care

The prevalence of obesity is rising and according to NCHS data more than one third of adults (78 million) are obese as defined by a body mass index (BMI) > 30 [6]. The obesity epidemic has led to a rise in the prevalence of OSA. In an epidemiologic study, Young et al. estimated the prevalence of OSA with an apnea-hypopnea index (AHI) of 15 or higher in patients aged 30–69 with a BMI > 40, to be 42–55% for men and 16–24% for women [7]. It is estimated that, between 1990 and 1998, there was a 12-fold increase in the diagnosis of OSA in surgical outpatients [8]. Some of the recent studies report a prevalence of >30% in neurosurgical patients and up to 91% in patients undergoing bariatric surgery [9]. In another series of 305 patients undergoing elective surgery and screened for OSA by the Berlin questionnaire about 24% patients were noted to be at risk of having OSA [10]. Similar estimates using the STOP (Snoring, Tiredness, Observed apneas, and high blood Pressure) questionnaire, revealed a prevalence of 27.5% in the presurgical population [11]. More recently using the NIS data (largest all payer inpatient discharge database sponsored by the AHRQ), Memtsoudis et al. reported a prevalence of sleep apnea (billed diagnosis) in 1998 versus 2007 to be 0.4% and 2.7% for general surgical procedures and 0.4% and 5.5% for orthopedic procedures, respectively [12].

### 2.2. Preoperative Assessment among Patients with Suspected OSA

With such high prevalence in the presurgical population a large majority of patients with OSA remain undiagnosed at the time of surgery. The gold standard for diagnosing and treating OSA is polysomnography (PSG); it may however not be practical for use in the preoperative setting in such a large population. Trying to identify patients at risk for OSA remains a challenge and surgical preoperative centers should have policies to help identify these patients resulting in appropriate triage and perioperative management. Screening should start with questions about daytime sleepiness, heavy snoring and sudden awakenings with need to catch breath, and witnessed apnea by a partner. Physical exam may provide additional clues like BMI > 30, short, thick neck, narrow oropharynx, tonsillar hypertrophy, and retrognathia. Among the available screening tools are an 11-point scoring instrument named the Berlin Questionnaire and the STOP and STOP-BANG (B, BMI > 35; A, age > 50 years; N, neck circumference > 40 cm, and G, male gender) questionnaire which is much easier to use. Pulse oximetry is more widely available and cheap; however, its sensitivity and specificity vary widely in studies [13–15]. The standard preoperative assessment in patients undergoing bariatric surgery includes a formal sleep evaluation using PSG and preoperative initiation of continuous positive airway pressure (CPAP) and titration.

### 2.3. Perioperative Outcomes in Patients with OSA Undergoing Noncardiac Surgery

The earliest cases of postoperative complications like respiratory failure among patients with OSA undergoing surgery unrelated to the treatment of OSA were reported by Rennotte et al. in 1995 suggesting that nasal CPAP used before surgery and resumed immediately after extubation helped reduce postoperative complications in patients with OSA [16]. Shortly thereafter in 1997, Ostermeier et al., reported three cases of sudden postoperative respiratory arrest associated with epidural opioids in patients with sleep apnea [17]. All three patients died. The first ever case control study by Gupta et al. using PSG and pulse oximetry data for OSA diagnosis in 101 patients undergoing orthopedic surgery, found a statistically significant higher incidence of postoperative serious complications (listed as unplanned ICU days, reintubations, and cardiac events, 24% versus 9%, *P* = 0.004) and hospital length of stay (6.8 days versus 5.1 days, *P* < 0.007) [18]. Other similar studies gave varied results in reporting postoperative outcomes among patients with OSA undergoing noncardiac surgery. Among many reasons for such variations in reporting the postoperative complications between different studies are whether OSA was diagnosed by clinical screening or by a gold standard test and especially among the case control studies whether the comparison was against a group of “true controls”; that is, OSA was excluded by formal PSG. Taking these limitations into consideration, to separate cases from true controls, our group used PSG data on all 282 patients undergoing noncardiac surgery. This study confirmed that patients with OSA had a higher incidence of postoperative hypoxemia (OR, 7.9; *P* = 0.009), overall complications (OR, 6.9; *P* = 0.003), unplanned ICU transfer (OR, 4.43; *P* = 0.069), and higher hospital length of stay (OR, 1.65; *P* = 0.049) compared to 189 controls [19]. Also to account for study limitations as well as the single center data reported in most of these studies our group reported a meta-analysis using data on 3942 patients from 13 case control or cohort studies [20]. We found a significant increase in the incidence of desaturation (OR, 2.27; *P* = 0.01), intensive care transfer (OR, 2.81; *P* = 0.002), respiratory failure (OR, 2.43; *P* = 0.003), and cardiac events (OR, 2.07; *P* = 0.007) in patients with OSA.

More recently a large multicenter, retrospective, case controlled study using the NIS database on patients undergoing orthopedic and general surgery reported a higher incidence pulmonary complications (aspiration pneumonia: 1.18% versus 0.84% and 2.79% versus 2.05%; ARDS 1.06% versus 0.45% and 3.79% versus 2.44%; intubation/mechanical ventilation 3.99% versus 0.79% and 10.8% versus 5.94%, all *P* values < 0.0001) in OSA patients compared to controls in both the surgery groups [12]. Another study using the NIS database confirmed that sleep disordered breathing was associated with increased odds for emergent intubation and mechanical ventilation in patients undergoing orthopedic surgery (OR 14.3, 95% CI 13.3–15.3, *P* < 0.001), prostate surgery (OR 10.3, 95% CI 8–13.3, *P* < 0.001), abdominal surgery (OR 2.01, 95% CI 1.7–2.4), and cardiac surgery (OR 1.8, 95% CI 1.65–1.95, *P* < 0.001). It was also associated with increased odds for atrial fibrillation in all the groups. This study also looked at mortality and found that OSA was independently associated with decreased mortality in the orthopedic (OR 0.65, 95% CI 0.45–0.95, *P* = 0.03), abdominal (OR 0.38, 95% CI 0.22–0.65; *P* = 0.001), and cardiovascular (OR 0.54, 95% CI 0.40–0.73; *P* < 0.001) cohorts; it had no impact on mortality in the prostate surgery cohort [21]. One way in which the authors explain this result was that patients with OSA have a better prognosis after postoperative respiratory failure compared to those without OSA but the lower overall mortality is not explained. Both these studies have limitations inherent to using administrative databases, wherein the diagnosis is taken from coding and billing data only. For the same reason hospital readmission rates could not be evaluated in either of these studies.

### 2.4. Perioperative Risk Reduction Strategies in Obstructive Sleep Apnea

Patients with OSA present with difficult intubation and airway management issues. Both anesthesia and sleep induce reductions in pharyngeal dilator muscle activation and lung volume, thereby predisposing to upper airway obstruction [22]. Patients with known OSA using CPAP at home should be advised to bring their machine to the hospital at the preoperative visit. Patients who are noncompliant should be encouraged to use it more consistently. In patients with previously undiagnosed OSA and high clinical suspicion of OSA based on preoperative assessment, if significant use of opioids and sedation is anticipated based on the type of the surgical procedure, consideration should be given to inpatient surgery as opposed to ambulatory surgery. Wherever possible regional anesthesia and blocks are preferred and for general anesthesia short acting agents like desflurane, propofol and succinylcholine are recommended. A recent multicenter study on sleep apnea patients undergoing total joint arthroplasty found that patients undergoing surgery under neuraxial anesthesia had significant lower rates of major complications compared in which combined neuraxial and general or general anesthesia was used [23]. When using general anesthesia the possibility of difficult intubation should be kept in mind and preparation for induction and intubation should follow the ASA difficult airway guidelines [24]. Full reversal of neuromuscular blockade (when applicable) using a nerve stimulator should be verified prior to extubation [25]. These patients must be fully awake, normothermic, hemodynamically stable, spontaneously breathing with and adequate respiratory rate, and tidal volume prior to breathing.

Multimodal analgesia involving the use of NSAIDS, acetaminophen, tramadol, ketamine, pregabalin, and COX-2 inhibitors should be considered to minimize the use of opioids in the postoperative setting. OSA patients receiving opioids are more likely than patients receiving nonopioid analgesia to have perioperative oxygen desaturations [26]. Patients undergoing ambulatory surgeries should be observed for an extended period of time prior to their discharge home [27]. Continuous pulse oximetry monitoring is recommended in the postoperative setting in patients at risk for respiratory complications of OSA. Cautious use of supplemental oxygen is advised after extubation until the patient is able to maintain his/her baseline oxygen saturation on room air. If frequent or severe airway obstruction or hypoxemia is noted in the postoperative monitoring, consideration should be made to initiate CPAP of NIPPV.

Conclusive evidence mandating perioperative use of CPAP among patients not currently using it is still lacking. A 16% absolute risk reduction in the rate of respiratory failure was reported in a bariatric surgery population with the use of noninvasive ventilation during the first 48 hours after extubation [28]. Among patients with OSA cardioverted for atrial fibrillation, those treated with CPAP were less likely to develop recurrent atrial fibrillation within 12 months of cardioversion compared to patients without treatment with CPAP [29]. A recent study randomized patients with high sleep apnea clinical score (SACS) undergoing elective orthopedic surgery to standard care with auto-PAP and standard care. No differences in length of stay (*P* = 0.65) or secondary outcomes (including unplanned ICU transfer, arrhythmia, MI, or delirium) were observed between the two groups [30].

## 3. Obesity Hypoventilation Syndrome

### 3.1. Importance of Preoperative Screening and Perioperative Implications

Obesity hypoventilation syndrome represents an embedded epidemic of highly morbid patients within the obesity and OSA epidemics. The prevalence of obesity hypoventilation syndrome (OHS) is also rising with the obesity epidemic and it is important for anesthesiologists to recognize and manage these patients perioperatively. A recent meta-analysis of a cohort of more than 4000 patients with OSA reported a 19% prevalence of OHS confirming an overall prevalence of approximately 3 per 1000 [31]. Among OSA patients the prevalence of OHS is reported as 11% and about 90% of patients with OHS have OSA [32]. Studies on bariatric surgery patients have estimated the prevalence of OHS from 7% to 22%, with an overall prevalence of about 8% [33–35].

OHS is characterized by a triad of chronic daytime hypercapnia (PaCO_2_ ≥ 45 mm Hg), sleep disordered breathing and obesity with a BMI > 30 kg/m^2^ [20, 21]. Arterial blood gas (ABG) measurements are important for confirming chronic daytime diurnal hypercapnia; however, these often cannot be obtained in routine outpatient preoperative settings. Moreover, the diagnosis of OHS can only be established after excluding other potential causes of hypercapnia like severe obstructive airway disease, severe chest wall deformities like kyphoscoliosis, severe interstitial lung disease, and neuromuscular disorders. Given these practical difficulties in establishing the diagnosis there are yet no studies reporting postoperative outcomes among patients with OHS. Patients with OHS tend to have high serum bicarbonate as a metabolic compensation for chronic respiratory acidosis. High serum bicarbonate combined with severity of OSA has been suggested to screen patients for OHS preoperatively [36]. The diagnosis should be confirmed with ABG in suspected OHS patient with high serum bicarbonate (>27 meq/L).

The general principles of perioperative management in patients with OHS are similar to those in OSA, keeping in mind that patients with OHS tend to represent a sicker cohort. Mortality as high as 23% has been reported in untreated patients with OHS compared to 9% in well-matched obese cohorts at 1.5 years after hospital discharge, adjusted HR of 4.0 (CI: 1.5–10.4) [37]. Untreated patients with OHS are also at risk for pulmonary artery hypertension secondary to chronic hypoxemia and right-sided heart failure [37, 38]. Patients with OHS have blunting of their respiratory drive in response of hypercapnia compared to eucapnic obese patients [24, 25]. A recent retrospective study showed that most patients with OHS are unrecognized at the time of elective surgery and among patients with OSA; those having OHS are at the highest risk of respiratory failure after elective surgery (44.4% versus 2.6%) [39]. In patients suspected to have OHS and those with known OHS where PAP setting is not known as an empiric inspiratory positive airway pressure (PAP) of 16–18 cm H_2_O and expiratory PAP of 9-10 cm H_2_O can be initiated to overcome upper airway obstruction and improve ventilation [40].

## 4. Pulmonary Hypertension

### 4.1. Pathophysiology of Postoperative Complications in Pulmonary Hypertension

Pulmonary artery pressure is a function of left atrial pressure, cardiac output, and pulmonary vascular resistance (PVR). PH may be potentially worsened by several factors during the perioperative period for, for example, hypoxia, acidosis, hypercapnia, positive pressure ventilation, and acute lung injury which may work to increase PVR. Cement, bone marrow, or air embolization to the pulmonary vasculature during hip replacement surgery may also lead to rapid rises in PH. Severe PH and exacerbation of moderate pulmonary hypertension can result in RV failure and acute cardiogenic shock. In normal individuals RV perfusion occurs both in systole and diastole. In patients with PH, as RVSP rises and approaches systemic blood pressure, it leads to compromise in systolic coronary blood flow to the RV. Failing RV leads to increase in RV end diastolic pressures limiting diastolic perfusion as well [41], setting up the cycle for RV ischemia, and further worsening RV function, leading to a decrease in cardiac output and eventually cardiogenic shock.

### 4.2. Perioperative Outcomes in Patients with Pulmonary Hypertension Undergoing Noncardiac Surgery

Pulmonary hypertension is defined by the WHO as mean pulmonary arterial pressure (MPAP) >25 mm Hg at rest or >30 mm Hg during exercise. It has long been recognized as an independent risk factor for perioperative complications after cardiac surgery [42, 43], with mortality rates of up to 25% [44]. However, PH is not yet included as an independent risk factor for postoperative complications in patients undergoing noncardiac surgery by the ACC/AHA practice guidelines for noncardiac surgery. In a study on 145 patients undergoing noncardiac surgery, Ramakrishna et al. reported a 30-day mortality rate of 7%, and a rate of 42% for one or more short-term morbid event(s) (1.8 events/patient experiencing any event). Respiratory failure (28%), cardiac dysrhythmia (12%), and congestive heart failure (11%) were the most frequent postoperative morbid events. This study also helped define some measures of perioperative risk among patients with PH. These are right axis deviation on EKG, echocardiographic criteria (right ventricular hypertrophy, RV index of myocardial performance ≥0.75), RVSP (right ventricular systolic pressure)/systolic blood pressure ≥0.66, and anesthesia when nitrous oxide was not used [45]. Using multicenter data Memtsoudis et al., reported patients with PH undergoing total hip arthroplasty had an approximately 4-fold increased adjusted risk of mortality, and those undergoing total knee arthroplasty a 4.5-fold increased adjusted risk of mortality compared with patients without PH in the matched sample (*P* < 0.001 for each comparison) [46]. In the first ever case control study using right heart catheterization (RHC) data among all patients who underwent any elective noncardiac surgery subsequently, our group confirmed that patients with PH were also more likely to develop congestive heart failure (*P* < 0.001; OR: 11.9), hemodynamic instability (*P* < 0.002), sepsis (*P* < 0.0005), and respiratory failure (*P* < 0.004) [47]. Patients with PH also had longer ICU stay (*P* < 0.04), higher 30-day readmission rate (*P* < 0.008), and longer time on mechanical ventilation (*P* < 0.002). Mean pulmonary artery pressure was identified as an independent risk factor for postoperative morbidity. More recently Meyer et al. in a prospective observational study on 114 patients reported the overall mortality rates in PH patients undergoing major noncardiac surgery, nonobstetric surgery to be around 3.5%. The mortality rate for emergency surgery was reported to be 15% [48].

### 4.3. Perioperative Risk Reduction Strategies in Patients with Pulmonary Hypertension

**Figure 1:**
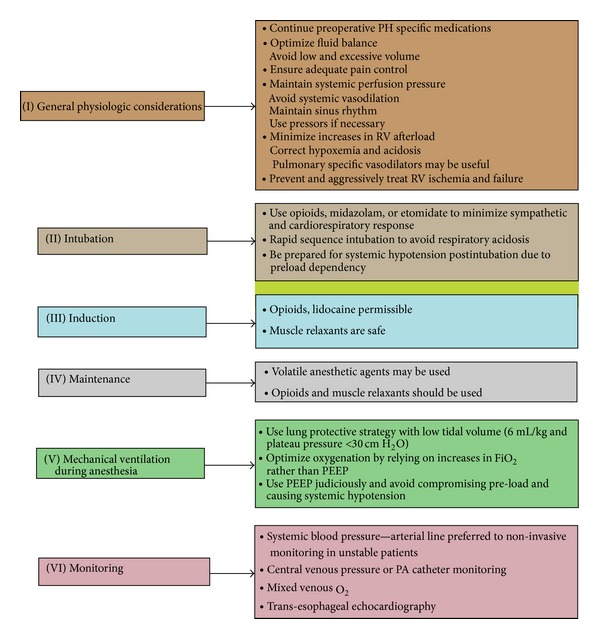
Intraoperative and postoperative management in PH patients with known PH. PA: pulmonary artery (adapted from reference [49]).

Preoperative evaluation of PH patients undergoing noncardiac surgery should include evaluating the severity and cause of PH (Figure 1) [49]. Wherever necessary the severity of PH should be evaluated by way of RHC and concomitant left heart catheterization when left-sided valvular disease or CAD is suspected or the accuracy of pulmonary artery wedge pressure is in question. Perioperative PA catheter monitoring offers several advantages like accurate measurement of PAP (to guide vasodilator therapy), cardiac output (CO), mixed venous oxygen saturation, calculated PVR, CVP, and pulmonary capillary wedge pressure (PCWP) (to guide need for fluids and vasopressors). Angiographic elevations of right atrial pressure and decrease in the cardiac output are of much greater consequence than the elevation of pulmonary artery pressure as these indicate right ventricular (RV) failure [49]. Once confirmed the need for elective surgery in patients with RV failure should be reevaluated and any surgery other than minor surgery should be avoided in these patients. In less-severe cases, or if elevated RA pressure and decreased CO are noticed during surgery; inotropic vasopressors can be tried to treat hypotension and if improved pulmonary vasodilators can then be initiated to decrease PVR [49]. TEE probe insertion after anesthesia induction may be helpful to assess PAP and RV size/function among patients with PH undergoing prolonged surgery or higher anticipated blood loss but has the same hemodynamic effects that follow endotracheal intubation and hence caution is advised [49].

General anesthesia is the most commonly used especially in patients with prolonged surgical time and spinal anesthesia is usually avoided due to its rapid onset and profound sympatholytic effect. No studies have compared the use of general versus regional anesthesia in PH patients. Anesthetic agents with minimal effects on myocardial contractility and SVR like etomidate are preferred while propofol and sodium pentothal having the exact opposite effects should be avoided for induction [50, 51]. During anesthesia systemic vasodilators should be avoided and adequate pain control should be ensured. Oxygenation during mechanical ventilation should rely on FiO_2_ rather than positive end expiratory pressure (PEEP).

Patients on PH specific therapy should continue this without interruption. If initiation is required for the first time, short acting pulmonary vasodilators like inhaled nitric oxide or prostacyclin or oral/parenteral sildenafil are recommended [52, 53]. Life threatening rebound PH should be anticipated in patients being weaned off from pulmonary vasodilators especially nitric oxide [54]. Gradual weaning and introduction of sildenafil (among patients on NO) may help [55]. Patients on anticoagulation for PH can interrupt this without bridging. Avoidance of systemic hypotension, hypo or hypervolemia, hypoxia, hypercarbia, hypothermia, and acidosis are key factors during the maintenance of anesthesia in these patients. Refractory hypotension can be managed by prompt intra-aortic balloon counter pulsation, LV assist device, or extracorporeal membrane oxygenation [56] when and where appropriate. RV assist devices can have harmful effects on pulmonary microcirculation and atrial septostomy can be used as a bridge to lung transplant or for palliation [56].

## References

[1] Rosen AK, Geraci JM, Ash AS, McNiff KJ, Moskowitz MA (1992). Postoperative adverse events of common surgical procedures in the Medicare population. *Medical Care*.

[2] Escarce JJ, Shea JA, Chen W, Qian Z, Schwartz JS (1995). Outcomes of open cholecystectomy in the elderly: a longitudinal analysis of 21,000 cases in the prelaparoscopic era. *Surgery*.

[3] Pedersen T (1994). Complications and death following anaesthesia. A prospective study with special reference to the influence of patient-, anaesthesia-, and surgery-related risk factors. *Danish Medical Bulletin*.

[4] Smetana GW, Lawrence VA, Cornell JE (2006). Preoperative pulmonary risk stratification for noncardiothoracic surgery: systematic review for the American College of Physicians. *Annals of Internal Medicine*.

[5] Qaseem A, Snow V, Fitterman N (2006). Risk assessment for and strategies to reduce perioperative pulmonary complications for patients undergoing noncardiothoracic surgery: a guideline from the American College of Physicians. *Annals of Internal Medicine*.

[6] Ogden CL, Lamb MM, Carroll MD, Flegal KM (2012). Obesity and socioeconomic status in adults: United States, 2005–2008. *NCHS Data Brief*.

[7] Young T, Peppard PE, Taheri S (2005). Excess weight and sleep-disordered breathing. *Journal of Applied Physiology*.

[8] Namen AM, Dunagan DP, Fleischer A (2002). Increased physician-reported sleep apnea: the National Ambulatory Medical Care survey. *Chest*.

[9] Hallowell PT, Stellato TA, Schuster M (2007). Potentially life-threatening sleep apnea is unrecognized without aggressive evaluation. *American Journal of Surgery*.

[10] Chung F, Ward B, Ho J, Yuan H, Kayumov L, Shapiro C (2007). Preoperative identification of sleep apnea risk in elective surgical patients, using the Berlin questionnaire. *Journal of Clinical Anesthesia*.

[11] Chung F, Yegneswaran B, Liao P (2008). STOP questionnaire: a tool to screen patients for obstructive sleep apnea. *Anesthesiology*.

[12] Memtsoudis S, Liu SS, Ma Y (2011). Perioperative pulmonary outcomes in patients with sleep apnea after noncardiac surgery. *Anesthesia and Analgesia*.

[13] Yamashiro Y, Kryger MH (1995). Nocturnal oximetry: is it a screening tool for sleep disorders?. *Sleep*.

[14] Lévy P, Pépin JL, Deschaux-Blanc C, Paramelle B, Brambilla C (1996). Accuracy of oximetry for detection of respiratory disturbances in sleep apnea syndrome. *Chest*.

[15] Whitelaw WA, Brant RF, Flemons WW (2005). Clinical usefulness of home oximetry compared with polysomnography for assessment of sleep apnea. *American Journal of Respiratory and Critical Care Medicine*.

[16] Rennotte M-T, Baele P, Aubert G, Rodenstein DO (1995). Nasal continuous positive airway pressure in the perioperative management of patients with obstructive sleep apnea submitted to surgery. *Chest*.

[17] Ostermeier AM, Roizen MF, Hautkappe M, Klock PA, Klafta JM (1997). Three sudden postoperative respiratory arrests associated with epidural opioids in patients with sleep apnea. *Anesthesia and Analgesia*.

[18] Gupta RM, Parvizi J, Hanssen AD, Gay PC (2001). Postoperative complications in patients with obstructive sleep apnea syndrome undergoing hip or knee replacement: a case-control study. *Mayo Clinic Proceedings*.

[19] Kaw R, Pasupuleti V, Walker E, Ramaswamy A, Foldvary-Schafer N (2012). Postoperative complications in patients with obstructive sleep apnea. *Chest*.

[20] Kaw R, Chung F, Pasupuleti V, Mehta J, Gay PC, Hernandez AV (2012). Meta-analysis of the association between obstructive sleep apnoea and postoperative outcome. *British Journal of Anaesthesia*.

[21] Mokhlesi B, Hovda MD, Vekhter B, Arora VM, Chung F, Meltzer DO (2013). Sleep-disordered breathing and postoperative outcomes after elective surgery: analysis of the Nationwide Inpatient Sample. *Chest*.

[22] Hillman DR, Platt PR, Eastwood PR (2010). Anesthesia, sleep, and upper airway collapsibility. *Anesthesiology Clinics*.

[23] Memtsoudis SG, Stundner O, Rasul R (2013). Sleep apnea and total joint arthroplasty under various types of anesthesia: a population-based study of perioperative outcomes. *Regional Anesthesia and Pain Medicine*.

[24] Rosenblatt WH, Whipple J (2003). The difficult airway algorithm of the American Society of Anesthesiologists. *Anesthesia and Analgesia*.

[25] Murphy GS, Szokol JW, Marymont JH, Greenberg SB, Avram MJ, Vender JS (2008). Residual neuromuscular blockade and critical respiratory events in the postanesthesia care unit. *Anesthesia and Analgesia*.

[26] Bolden N, Smith CE, Auckley D, Makarski J, Avula R (2007). Perioperative complications during use of an obstructive sleep apnea protocol following surgery and anesthesia. *Anesthesia and Analgesia*.

[27] Gross JB, Bachenberg KL, Benumof JL (2006). Practice guidelines for the perioperative management of patients with obstructive sleep apnea: a report by the American Society of Anesthesiologists Task Force on Perioperative Management of patients with obstructive sleep apnea. *Anesthesiology*.

[28] El Solh AA, Aquilina A, Pineda L, Dhanvantri V, Grant B, Bouquin P (2006). Noninvasive ventilation for prevention of post-extubation respiratory failure in obese patients. *European Respiratory Journal*.

[29] Kanagala R, Murali NS, Friedman PA (2003). Obstructive sleep apnea and the recurrence of atrial fibrillation. *Circulation*.

[30] O’gorman SM, Gay PC, Morgenthaler TI (2013). Does autotitrating positive airway pressure therapy improve postoperative outcome in patients at risk for obstructive sleep apnea syndrome?: a randomized controlled clinical trial. *Chest*.

[31] Kaw R, Hernandez AV, Walker E, Aboussouan L, Mokhlesi B (2009). Determinants of hypercapnia in obese patients with obstructive sleep apnea: a systematic review and metaanalysis of cohort studies. *Chest*.

[32] Mokhlesi B, Tulaimat A, Faibussowitsch I, Wang Y, Evans AT (2007). Obesity hypoventilation syndrome: prevalence and predictors in patients with obstructive sleep apnea. *Sleep and Breathing*.

[33] Lecube A, Sampol G, Lloberes P (2010). Asymptomatic sleep-disordered breathing in premenopausal women awaiting bariatric surgery. *Obesity Surgery*.

[34] Sugerman HJ, Fairman RP, Baron PL, Kwentus JA (1986). Gastric surgery for respiratory insufficiency of obesity. *Chest*.

[35] Dominguez-Cherit G, Gonzalez R, Borunda D, Pedroza J, Gonzalez-Barranco J, Herrera MF (1998). Anesthesia for morbidly obese patients. *World Journal of Surgery*.

[36] Mokhlesi B, Tulaimat A, Faibussowitsch I, Wang Y, Evans AT (2007). Obesity hypoventilation syndrome: prevalence and predictors in patients with obstructive sleep apnea. *Sleep and Breathing*.

[37] Nowbar S, Burkart KM, Gonzales R (2004). Obesity-associated hypoventilation in hospitalized patients: prevalence, effects, and outcome. *American Journal of Medicine*.

[38] Kessler R, Chaouat A, Weitzenblum E (1996). Pulmonary hypertension in the obstructive sleep apnoea syndrome: prevalence, causes and therapeutic consequences. *European Respiratory Journal*.

[39] Kaw R, Pasupuleti V, Walker E Obesity hypoventilation syndrome: an emerging and unrecognized risk factor among surgical patients.

[40] Chau EH, Lam D, Wong J, Mokhlesi B, Chung F (2012). Obesity hypoventilation syndrome: a review of epidemiology, pathophysiology, and perioperative considerations. *Anesthesiology*.

[41] Vlahakes GJ (1996). Management of pulmonary hypertension and right ventricular failure: another step forward. *The Annals of Thoracic Surgery*.

[42] Kuralay E, Demírkiliç U, Öz BS, Cíngöz F, Tatar H (2002). Primary pulmonary hypertension and coronary artery bypass surgery. *Journal of Cardiac Surgery*.

[43] Beck JR, Mongero LB, Kroslowitz RM (1999). Inhaled nitric oxide improves hemodynamics in patients with acute pulmonary hypertension after high-risk cardiac surgery. *Perfusion*.

[44] Reich DL, Bodian CA, Krol M, Kuroda M, Osinski T, Thys DM (1999). Intraoperative hemodynamic predictors of mortality, stroke, and myocardial infarction after coronary artery bypass surgery. *Anesthesia and Analgesia*.

[45] Ramakrishna G, Sprung J, Ravi BS, Chandrasekaran K, McGoon MD (2005). Impact of pulmonary hypertension on the outcomes of noncardiac surgery: Predictors of perioperative morbidity and mortality. *Journal of the American College of Cardiology*.

[46] Memtsoudis SG, Ma Y, Chiu YL, Walz JM, Voswinckel R, Mazumdar M (2010). Perioperative mortality in patients with pulmonary hypertension undergoing major joint replacement. *Anesthesia and Analgesia*.

[47] Kaw R, Pasupuleti V, Deshpande A, Hamieh T, Walker E, Minai OA (2011). Pulmonary hypertension: an important predictor of outcomes in patients undergoing non-cardiac surgery. *Respiratory Medicine*.

[48] Meyer S, Mclaughlin VV, Seyfarth HJ (2013). Outcomes of noncardiac, nonobstetric surgery in patients with PAH: an international prospective survey. *European Respiratory Journal*.

[49] Minai OA, Yared JP, Kaw R, Subramaniam K, Hill NS (2013). Perioperative risk and management in patients with pulmonary hypertension. *Chest*.

[50] Sarkar M, Laussen PC, Zurakowski D, Shukla A, Kussman B, Odegard KC (2005). Hemodynamic responses to etomidate on induction of anesthesia in pediatric patients. *Anesthesia and Analgesia*.

[51] Ebert TJ, Muzi M, Berens R, Goff D, Kampine JP (1992). Sympathetic responses to induction of anesthesia in humans with propofol or etomidate. *Anesthesiology*.

[52] Slomka F, Salmeron S, Zetlaoui P, Cohen H, Simonneau G, Samii K (1988). Primary pulmonary hypertension and pregnancy: anesthetic management for delivery. *Anesthesiology*.

[53] Dunbar Ivy D, Griebel JL, Kinsella JP, Abman SH (1998). Acute hemodynamic effects of pulsed delivery of low flow nasal nitric oxide in children with pulmonary hypertension. *Journal of Pediatrics*.

[54] Ivy DD, Kinsella JP, Ziegler JW, Abman SH (1998). Dipyridamole attenuates rebound pulmonary hypertension after inhaled nitric oxide withdrawal in postoperative congenital heart disease. *Journal of Thoracic and Cardiovascular Surgery*.

[55] Andrew MA, Wessel DL (1999). Sildenafil ameliorates effects of inhaled nitric oxide withdrawal. *Anesthesiology*.

[56] Keogh AM, Mayer E, Benza RL (2009). Interventional and surgical modalities of treatment in pulmonary hypertension. *Journal of the American College of Cardiology*.

